# The Role of HBx Mutations in Chronic Hepatitis B with Acute Exacerbation

**DOI:** 10.3390/v17091223

**Published:** 2025-09-07

**Authors:** Xiaobei Chen, Jinzhi Shi, Ping Zhou, Yunyun Tian, Yajing Zheng, Tingting Liu, Yan Li, Fan Zhu

**Affiliations:** 1 Department of Infectious Diseases, East Campus, Renmin Hospital of Wuhan University, Wuhan 430223, China; jinzhi@whu.edu.cn (J.S.); rm001850@whu.edu.cn (Y.T.); 2 State Key Laboratory of Virology and Biosafety, Department of Medical Microbiology, School of Basic Medical Sciences, Wuhan University, Wuhan 430071, China; zhouping@whu.edu.cn; 3 Department of Fever Clinic, East Campus, Renmin Hospital of Wuhan University, Wuhan 430223, China; zhengyajing@whu.edu.cn (Y.Z.); liutingtingting@whu.edu.cn (T.L.); zgcqlyy@163.com (Y.L.); 4 Hubei Province Key Laboratory of Allergy & Immunology, Wuhan University, Wuhan 430071, China

**Keywords:** chronic hepatitis B, acute exacerbation, HBV-related liver failure, X protein, mutation

## Abstract

Hepatitis B virus (HBV) infection remains a significant global health burden, primarily due to its chronic complications, including acute exacerbation, cirrhosis, hepatocellular carcinoma (HCC), and related sequelae. Acute exacerbation of chronic hepatitis B (CHB-AE) is common and often represents the earliest clinical manifestation. The Hepatitis B virus X protein (HBx) (17-kDa) is not only essential for viral replication but also plays a role in the development of HCC. To investigate the role of HBx mutation in CHB-AE progression, we enrolled 33 hospitalized CHB-AE patients and 31 patients with HBV-related liver failure (controls) from mainland China between January 2017 and June 2018. Single mutation 36 of HBx was significantly more prevalent in CHB-AE patients (*p* < 0.05), whereas Joint Mutation 1 was more frequent in HBV-related liver failure patients (*p* < 0.05). HBx mutations, including Single mutation 36 and Joint Mutations 2 and 3, were significantly associated with high HBV DNA levels (*p* < 0.05), while Joint mutation 1 predominated in the low HBV DNA group (*p* < 0.01). Age-stratified analysis showed that Single mutation 36 and Joint Mutation 2 were more common in younger patients (<35 years old) (*p* < 0.05), whereas Joint mutation 1 was more frequent in older age (≥35 years old) (*p* < 0.05). Moreover, antiviral therapy markedly reduced the prevalence of Joint mutation 1 from 82.98% in treatment-naïve patients to 29.41% in treatment-experienced patients (*p* < 0.0001). These findings suggest that specific HBx mutations are associated with viral replication levels, disease progression, and patient demographics. Such mutations may serve as molecular markers for disease severity and potential therapeutic targets in both CHB-AE and HBV-related liver failure.

## 1. Introduction

Hepatitis B Virus (HBV) infection continues to pose a major global health challenge. According to the World Health Organization (WHO), an estimated 254 million people were living with chronic hepatitis B infection in 2022, with 1.2 million new infections annually. In the same year, HBV-related complications such as cirrhosis and hepatocellular carcinoma (HCC) led to approximately 1.1 million deaths worldwide [1]. Current antiviral therapies, including pegylated interferon and nucleos(t)ide analogues (NAs), can slow disease progression and reduce the risk of cirrhosis and HCC, but they rarely achieve complete HBV eradication or hepatitis B surface antigen (HBsAg) clearance [2]. Chronic HBV infection (CHB) is a dynamic condition marked by complex interactions between the virus, hepatocytes, and the host immune system [3]. A characteristic clinical event is acute exacerbation (CHB-AE), which occurs in 10–30% of patients annually [4,5]. In HBV endemic regions, CHB-AE is common and often represents the earliest clinical manifestation of the disease [6]. It arises from immune clearance phase flares or viral reactivation in inactive or resolved carriers, potentially leading to severe liver injury [6,7]. Clinically, CHB-AE can manifest as elevated alanine aminotransferase (ALT), jaundice, and hepatic decompensation, with severe cases carrying a high mortality risk [8]. According to the 2015 Asian Pacific Association for the Study of the Liver (APASL) guideline, CHB-AE is defined as an intermittent ALT elevation > 5× the upper limit of normal and/or > 2× the baseline level [9]. The high burden of HBV infection and the challenge in managing CHB-AE emphasize the urgent need for better prognostic markers and therapeutic strategies.

The HBV genome is a partially double-stranded relaxed circular DNA (rcDNA) composed of four open reading frames (ORFs): preS1/S2/S, pre-core/core, polymerase, and X. These ORFs encode viral proteins, including the hepatitis B surface (HBs) antigens (LHBs, MHBs, and SHBs), hepatitis B e antigen (HBe), hepatitis B core antigen (HBc), DNA polymerase (DNA Pol), and Hepatitis B virus X protein (HBx) [10]. HBx, a 154–amino acid non-structural protein (~17 kDa) encoded by the X-ORF, is essential for HBV replication and modulation of host cellular functions [11,12,13]. Structurally, the N-terminal third (residues 1–50) of HBx functions as a negative regulator, while the C-terminal domain acts as a transcriptional activator [14]. In the nucleus, HBx interacts with covalently closed circular DNA (cccDNA) [15] and the transcriptional machinery to promote viral gene expression [16,17,18]. In the cytoplasm, it modulates signaling pathways that affect cell survival, metabolism, and proliferation [19,20]. These pleiotropic activities link HBx not only to HBV replication but also to hepatocarcinogenesis [21,22,23,24]. Due to the error-prone reverse transcription process in HBV replication, the viral genome—especially HBx—is prone to mutations, including point mutations, insertions, and deletions [25]. Such genetic variations can alter viral replication capacity, disease progression, and responses to antiviral therapies, occasionally leading to drug resistance [26].

However, the role of HBx mutations in CHB-AE remains poorly characterized. Clarifying this relationship could improve our understanding of HBV pathogenesis, help identify molecular markers of disease severity, and inform the development of more effective therapeutic strategies.

## 2. Materials and Methods

### 2.1. Sample Collection

A total of 64 serum samples were collected from patients at Renmin Hospital of Wuhan University between January 2017 and June 2018.

### 2.2. Exclusion Criteria

Patients were excluded from the study if they met any of the following conditions:

Positive for HIV antibody;

Positive for HAV antibody;

Positive for HCV antibody;

Positive for HDV antibody;

Negative or undetectable HBV DNA levels in serum.

### 2.3. Serologic Testing

HBV serological markers were determined by using the chemiluminescent microparticle immunoassay (CMIA) technique with an Architect-i2000 automatic analyzer (Abbott Laboratories, Chicago, IL, USA). Commercially available kits from Abbott Laboratories were used for testing. The analytical threshold of anti-HBs was set at 10 mIU/mL. Serum samples with HBsAg concentrations greater than 25,000 IU/mL were diluted according to the Manual Dilution Procedure. ALT levels were measured using an ADVIA automatic biochemical analyzer (Siemens, Munich, Germany).

### 2.4. HBV DNA Quantification

Blood samples were stored at −70 °C until processing. HBV DNA was extracted from 600 μL of serum and quantified using a commercially available real-time fluorescence quantitative kit (FOSUN DIAGNOSTICS, Shanghai, China) with a lower detection threshold of 15 IU/mL.

### 2.5. HBx Gene Amplification

The extracted HBV DNA was used as a template for polymerase chain reaction (PCR) to amplify the HBx gene. The primer sequences were:

Forward primer: 5′-ATGGCTGCTAGGCTGTGCTGCCAAC-3′.
Reverse primer: 5′-TTAGGCAGAGGTGAAAAAGTTGCAT-3′.

The PCR conditions were as follows:

(1) Initial denaturation at 95 °C for 5 min.
(2) 35 cycles of denaturation at 94 °C for 30 s, annealing at 55 °C for 30 s, and extension at 72 °C for 30 s.
(3) Final extension at 72 °C for 10 min.

The amplified products were separated on a 1.5% agarose gel prepared in 1× TAE buffer containing GelRed nucleic acid stain. Electrophoresis was performed at 100 V for 40 min, and bands were visualized under ultraviolet light.

### 2.6. HBx Mutation Analysis

The PCR products of the HBx gene were sequenced, and the resulting sequences were aligned with the HBV reference sequence (X01587.1) [27] available in the NCBI database using the BLAST+ 2.17.0 tool (https://blast.ncbi.nlm.nih.gov/Blast.cgi) accessed on 26 July 2025. Mutations were identified through this comparative analysis.

### 2.7. Statistical Analysis

Statistical analyses were performed using SPSS software (Version 20, IBM Corp., Armonk, NY, USA). Categorical variables were analyzed using the chi-square test, while continuous variables were compared using Student’s *t*-test. A *p*-value of < 0.05 was considered statistically significant.

## 3. Results

### 3.1. Baseline Characteristics of Patients

#### 3.1.1. Study Cohort Characteristics

**Figure 1:**
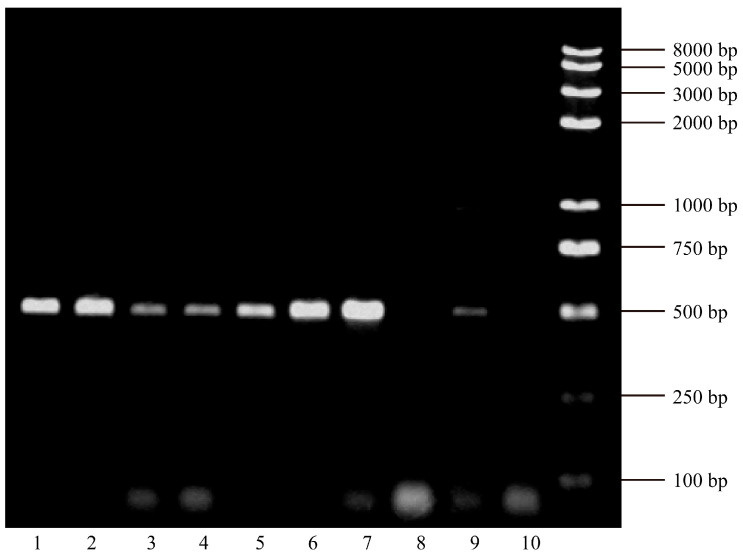
Electrophoretic analysis of HBx gene PCR products on an agarose gel. CHB-AE: Lane 1 (HBx 66 patient), 5 (HBx 57 patient), 7 (HBx 53 patient), 8 (HBx 49 patient) and 10 (HBx 22 patient); ACLF: Lane 2 (HBx 65 patient), 3 (HBx 62 patient), 4 (HBx 61 patient) and 9 (HBx 21 patient); Chronic liver failure: Lane 6 (HBx 56 patient).

**Figure 2:**
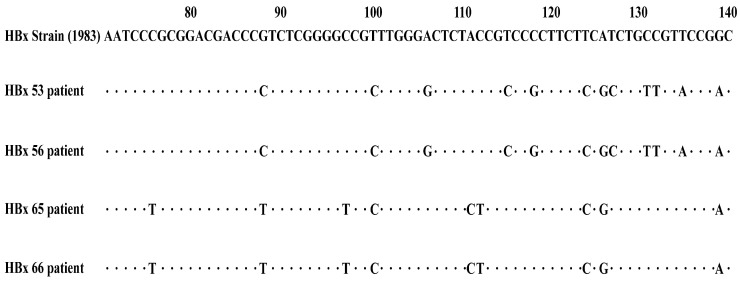
The HBx gene nucleotide sequence alignment of patients with CHB-AE and HBV-related liver failure.

Between January 2017 to June 2018, serum samples were collected from hospitalized patients at Renmin Hospital of Wuhan University. The study enrolled 33 patients with CHB-AE and 31 patients with HBV-related liver failure. The cohort consisted of 54 males and 10 females, with a mean age of 43.64 ± 12.85 years. Among liver failure cases, 25 had acute-on-chronic liver failure (ACLF), 5 had chronic liver failure, and 1 had acute liver failure. Diagnoses were made according to the “The guideline of prevention and treatment for chronic hepatitis B (2015 version)” [28] and the “Diagnostic and treatment guidelines for liver failure (2012 version)” [29]. The full-length HBx gene was successfully amplified from all samples, with an expected fragment size of approximately 465 bp (Figure 1). HBx Mutations were detected in all patients (64/64), with multiple mutation types observed at different loci (Figure 2).

#### 3.1.2. Baseline Comparison Between CHB-AE and HBV-Related Liver Failure

There were no significant differences between the two groups in gender distribution (CHB-AE: 78.79% male vs. liver failure: 90.32% male, *p* = 0.3546) or age (CHB-AE: 57.58% ≥ 35 years vs. liver failure: 70.97% ≥ 35 years, *p* = 0.2645).

However, CHB-AE patients had significantly higher HBV DNA levels (≥log 5 copies/mL: 87.88% vs. 64.52%, *p* = 0.0275) and higher HBeAg positivity (63.64% vs. 32.26%, *p* = 0.0121) (Table 1).

### 3.2. The HBx Mutations and the Severity of CHB

Single mutation 36 (T36A/S): More frequent in CHB-AE than liver failure patients (39.39% vs. 16.13%, *p* = 0.0386).

Joint Mutation 1 (R26C + P33S + P38S): More frequent in liver failure (80.65%) than CHB-AE (57.58%, *p* = 0.0466).

Joint Mutation 2 (S39P + P40A + S43P + A44L/V/F + Q87R/W/K/G): Detected in 36.36% (12/33) of CHB-AE vs. 16.13% (5/31) of liver failure patients, without statistical significance (*p* = 0.0670).

**Figure 3:**
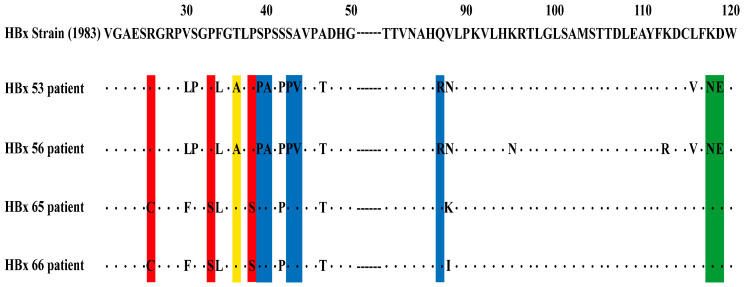
The HBx gene amino acid sequence alignment patients with CHB-AE and HBV-related liver failure. HBx53: patient 53#, CHB-AE, with Single mutation 36 (marked as yellow), joint 2 (marked as blue), and joint 3 (marked as green) mutations of HBx. HBx56: patient 56#, Chronic Liver Failure, with Single mutation 36, joint 2, and joint 3 mutations of HBx. HBx65: patient 65#, ACLF, with joint 1 (marked as red) mutation of HBx. HBx66: patient 66#, CHB-AE, with joint 1 mutation of HBx.

Joint Mutation 3 (K118N/T + D119E): No significant difference between two groups (33.33% vs. 16.13%, *p* = 0.1122) (Figure 3, Table 2).

Overall, the distribution of HBx mutations varies with the severity of CHB. Single mutation T36A/S was more prevalent in CHB-AE patients, whereas Joint Mutation 1 (R26C + P33S + P38S) occurred more frequently in liver failure cases. Joint Mutation 2 showed a higher occurrence in CHB-AE patients, but the difference was not statistically significant, while Joint Mutation 3 showed no significant difference between the two groups. These findings suggest that certain specific mutation patterns may be associated with different stages of disease progression, though further studies are needed for confirmation.

### 3.3. HBx Mutations and HBV DNA Viral Load

Joint Mutation 1 (R26C + P33S + P38S): More common in low viral load patients (100% vs. 59.18%, *p* = 0.0077). Joint Mutation 2 (S39P + P40A + S43P + A44L/V/F + Q87R/W/K/G): Present only in high viral load patients (34.69% vs. 0%, *p* = 0.0199). Joint Mutation 3 (K118N/T + D119E): More frequent in high viral load patients (32.65% vs. 0%, *p* = 0.0268).

Single Mutation 36 (T36A/S): Detected only in high viral load patients (≥log 5: 36.73% vs. < log 5: 0%, *p* = 0.0147).

Overall, Single Mutation 36, Joint Mutation 2, and Joint Mutation 3 were significantly associated with high HBV DNA levels (*p* < 0.05), whereas Joint Mutation 1 HBx was linked to low HBV DNA levels (*p* < 0.01) (Table 3).

### 3.4. The HBx Mutations and Patients’ Age

Single mutation 36 (T36A/S): More frequent in younger patients (<35 years: 43.48% vs. ≥ 35 years: 19.51%, *p* = 0.0407).

Joint mutation 1 (R26C + P33S + P38S): More common in older patients (≥35 years: 78.05% vs. < 35 years: 52.17%, *p* = 0.0321).

Joint mutation 2 (S39P + P40A + S43P + A44L/V/F + Q87R/W/K/G): More prevalent in younger patients (43.48% vs. 17.07%, *p* = 0.0217).

Joint mutation 3 (K118N/T + D119E): No significant age-related difference (*p* = 0.0505).

Age-stratified analysis revealed that Single mutation 36 and Joint Mutation 2 were significantly more prevalent in patients < 35 years old (*p* < 0.05), while Joint Mutation 1 was more common in those aged 35 years and older (*p* < 0.05). Joint Mutation 3 showed no significant age association (Table 4).

### 3.5. HBx Mutations and Antiviral Treatment History

All HBx mutation frequencies were lower in treatment-experienced patients, but only Joint mutation 1 showed a significant reduction (treatment-naïve: 82.98% vs. treatment-experienced: 29.41%, *p* < 0.0001) (Table 5).

### 3.6. Risk Factors Related to Prognosis

Gender, age, HBV DNA level, HBeAg, HBx mutations and antiviral treatment were not significantly associated with prognosis (*p* > 0.05) (Supplementary Table S1).

## 4. Discussion

HBx is a multifunctional 17-kDa protein essential for HBV replication and pathogenesis [30]. Mutations in HBx influence disease progression through several mechanisms:

(1) Oncogenesis and Disease Progression: Certain variants (e.g., I127T, V131I, and F132Y/I/R) are associated with progression from chronic liver disease to HCC [31,32].
(2) Transcriptional regulation: HBx modulates both viral and host gene expression, impacting cell cycle, proliferation, apoptosis, and immune signaling [33,34].
(3) Immune Modulation: HBx enhances pro-inflammatory cytokine expression, contributing to persistent hepatic inflammation and HCC risk [35].
(4) Genomic Instability: HBx promotes chromosomal instability, a hallmark of tumorigenesis [36].
(5) Fibrosis and Cirrhosis: Certain HBx mutations are linked to cirrhosis onset [37,38].

These findings underscore the multifaceted roles of HBx mutations in promoting liver disease progression, liver failure, cirrhosis, and HCC. In this study, we explored the specific association between HBx mutations and the progression of CHB-AE and HBV-related liver failure.

Our study revealed a striking contrast between Single Mutation 36 and Joint Mutation 1. Single mutation 36 was significantly more prevalent in CHB-AE (*p* = 0.0386), whereas Joint Mutation 1 was more common in HBV-related liver failure (*p* = 0.0466), suggesting divergent roles in disease progression.

The relationship between HBx mutations and viral replication observed in our study aligns with the known role of HBx in enhancing cccDNA transcription and supporting high-level replication [39,40,41,42]. Mutations such as Single mutation 36, Joint Mutation 2, and Joint 3 were enriched in high viral load group, consistent with an active replication phenotype. In contrast, the predominance of Joint mutation 1 in low viral load patients suggests it may emerge during viral suppression or disease decompensation, potentially reflecting a late-stage adaptation.

Single Mutation 36 was found to be associated with higher HBV DNA levels and younger age (<35 years), whereas Joint Mutation 1 correlated with lower HBV DNA levels and older age (≥35 years). This pattern may be explained by the fact that younger individuals are more likely to be in immune tolerance or immune clearance phases, characterized by high viral loads and positive HBeAg status. In contrast, Joint Mutation 1 predominated in older individuals with lower viral loads.

These findings are consistent with previous reports suggesting that HBx mutations tend to accumulate with age [43]. Yuen et al. further proposed that increased viral diversity is associated with older age, accompanied by lower levels of HBV-DNA, HBsAg and HBeAg [44]. Collectively, these observations emphasize the importance of considering age as a potential confounder factor when assessing the relationship between HBx mutation and HCC risk [45].

Interestingly, Joint Mutation 1 appeared to be mutually exclusive with other mutations, suggesting that it may arise through distinct evolutionary or functional pathways. This exclusivity highlights the need for further studies to clarify its biological significance and potential role in disease progression.

Antiviral therapy also influenced mutation prevalence. The frequency of Joint Mutation 1 was markedly reduced in treatment-experienced patients compared to treatment-naïve patients (29.41% vs. 82.98%, *p* < 0.0001), suggesting that sustained viral suppression may limit its emergence. Previous studies have shown that HBx mutations can develop during lamivudine or entecavir therapy, sometimes contributing to drug resistance [12,46]. However, in our cohort, other mutations did not differ significantly between treatment groups, indicating that some HBx variants may arise independently of antiviral exposure.

We also observed significantly higher HBV DNA levels and HBeAg positivity in CHB-AE compared to liver failure patients, supporting the notion that active replication and immune clearance are characteristic of CHB-AE, whereas lower viral loads may reflect advanced disease or impaired immune control. This aligns with studies linking high viral loads to increased HCC risk, even under antiviral therapy [47,48,49,50,51].

Limitations of this study include the relatively small sample size and the lack of longitudinal follow-up, which restricts conclusions about causality and mutation dynamics over time. Larger, prospective studies are needed to confirm these associations and elucidate the molecular mechanisms by which specific HBx mutations influence disease phenotype.

## 5. Conclusions

We identified several distinct HBx mutation patterns—Single Mutation 36, Joint Mutations 1, 2, and 3—with opposing associations for Single Mutation 36 and Joint Mutation 1. Single Mutation 36 may serve as a biomarker for CHB-AE, while Joint Mutation 1 may indicate HBV-related liver failure. These findings provide new insights into HBV pathogenesis and suggest that HBx mutations could serve as useful biomarkers or therapeutic targets in managing chronic HBV infection.

## Figures and Tables

**Table 1:** The baseline characteristics of patients with CHB-AE and HBV-related liver failure.

		Chronic Hepatitis B with Acute Exacerbation (*n* = 33) (%)	HBV-Related Liver Failure (*n* = 31) (%)	*p*-Value
Gender	Male	26 (78.79)	28 (90.32)	0.3546
	Female	7 (21.21)	3 (9.68)	
Age (years)	≥35	19 (57.58)	22 (70.97)	0.2645
	<35	14 (42.42)	9 (29.03)	
HBV DNA Log level (copies/mL)	≥5	29 (87.88)	20 (64.52)	0.0275
	<5	4 (12.12)	11 (35.48)	
HBeAg (0–1 COI)	positive	21 (63.64)	10 (32.26)	0.0121
	negative	12 (36.36)	21 (67.74)	

**Table 2:** The HBx mutations and the severity of CHB.

HBx Mutations	Chronic Hepatitis B with Acute Exacerbation (*n* = 33) (%)	HBV-Related Liver Failure (*n* = 31) (%)	*p*-Value
Single Mutation 36 (T36A/S)	13 (39.39)	5 (16.13)	0.0386
Joint Mutation 1 (R26C + P33S + P38S)	19 (57.58)	25 (80.65)	0.0466
Joint Mutation 2 (S39P + P40A + S43P + A44L/V/F + Q87R/W/K/G)	12 (36.36)	5 (16.13)	0.0670
Joint Mutation 3 (K118N/T + D119E)	11 (33.33)	5 (16.13)	0.1122

**Table 3:** The HBxAg mutations and HBV DNA viral load.

HBx Mutations	High HBV DNA Log Level ≥ 5 (*n* = 49) (%)	Low HBV DNA Log Level < 5 (*n* = 15) (%)	*p*-Value
Single Mutation 36 (T36A/S)	18 (36.73)	0 (0)	0.0147
Joint Mutation 1 (R26C + P33S + P38S)	29 (59.18)	15 (100)	0.0077
Joint Mutation 2 (S39P + P40A + S43P + A44L/V/F + Q87R/W/K/G)	17 (34.69)	0 (0)	0.0199
Joint Mutation 3 (K118N/T + D119E)	16 (32.65)	0 (0)	0.0268

**Table 4:** The HBx mutations and patients’ age.

HBx Mutations	Age ≥ 35 (*n* = 41) (%)	Age < 35 (*n* = 23) (%)	*p*-Value
Single Mutation 36 (T36A/S)	8 (19.51)	10 (43.48)	0.0407
Joint Mutation 1 (R26C + P33S + P38S)	32 (78.05)	12 (52.17)	0.0321
Joint Mutation 2 (S39P + P40A + S43P + A44L/V/F + Q87R/W/K/G)	7 (17.07)	10 (43.48)	0.0217
Joint Mutation 3 (K118N/T + D119E)	7 (17.07)	9 (39.13%)	0.0505

**Table 5:** The HBx mutations and antiviral treatment history.

HBx Mutations	Treatment-Naïve (*n* = 47) (%)	Treatment-Experienced (*n* = 17) (%)	*p*-Value
Single Mutation 36 (T36A/S)	14 (29.79)	4 (23.53)	0.8595
Joint Mutation 1 (R26C + P33S + P38S)	39 (82.98)	5 (29.41)	<0.0001
Joint Mutation 2 (S39P + P40A + S43P + A44L/V/F + Q87R/W/K/G)	14 (29.79)	3 (17.65)	0.5152
Joint Mutation 3 (K118N/T + D119E)	13 (27.66)	3 (17.65)	0.6240

## Data Availability

All data are available from the corresponding author upon request.

## Supplementary Materials

The following supporting information can be downloaded at: https://www.mdpi.com/article/10.3390/v17091223/s1, Table S1: The risk factors related to prognosis.

## Abbreviations

The following abbreviations are used in this manuscript:

ACLF	Acute-on-chronic liver failure
ALT	Alanine aminotransferase
cccDNA	Covalently closed circular DNA
CHB-AE	Acute exacerbation of chronic hepatitis B
HBV	Hepatitis B virus
HBx	Hepatitis B virus X protein
HCC	Hepatocellular carcinoma
NAs	Nucleos(t)ide analogues
ORFs	Open reading frames
