# Supplementary material for: The Role of HBx Mutations in Chronic Hepatitis B with Acute Exacerbation
Source: Viruses. 2025 Sep 7;17(9):1223. doi: 10.3390/v17091223 (PMC12474432; doi:10.3390/v17091223)
Supplement: Supplementary file 1 [file viruses-17-01223-s001.zip › viruses-3643186-supplementary.pdf]

Supplementary Table S1

Supplementary Table S1. The risk factors related to prognosis

	Total	Improvement (n = 58) (%)	Death (n = 6) (%)	P-value
Gender	Male	48 (82.76)	6 (100.00)	0.5782
	Female	10 (17.24)	0 (0.00)	
Age (years)	≥ 35	36 (62.07)	5 (83.33)	0.5575
	< 35	22 (37.93)	1 (16.67)	
HBV DNA Log level (copies/mL)	≥ 5	46 (79.31)	3 (50.00)	0.2682
	< 5	12 (20.69)	3 (50.00)	
HBeAg (0-1 COI)	positive	29 (50.00)	2 (33.33)	0.7274
	negative	29 (50.00)	4 (66.67)	
Mutation 36	yes	16 (27.59)	2 (33.33)	0.8581
	no	42 (72.41)	4 (66.67)	
Joint Mutation 1 26, 33, 38	yes	40 (68.97)	4 (66.67)	0.7286
	no	18 (31.03)	2 (33.33)	
Joint Mutation 2 39, 40, 43, 44, 87	yes	15 (25.86)	2 (33.33)	0.9275
	no	43 (74.14)	4 (66.67)	
Joint Mutation 3 118, 119	yes	14 (24.14)	2 (33.33)	> 0.9999
	no	44 (75.86)	4 (66.67)	
Antiviral treatment	naïve	43 (74.14)	4 (66.67)	0.9275
	experienced	15 (25.86)	2 (33.33)	
